# A neonatal murine model of coxsackievirus A4 infection for evaluation of vaccines and antiviral drugs

**DOI:** 10.1080/22221751.2019.1673135

**Published:** 2019-10-09

**Authors:** Zhenjie Zhang, Xingcheng Zhang, Michael J. Carr, Hong Zhou, Juan Li, Shaoqiong Liu, Tao Liu, Weijia Xing, Weifeng Shi

**Affiliations:** a Key Laboratory of Etiology and Epidemiology of Emerging Infectious Diseases in Universities of Shandong, Shandong First Medical University & Shandong Academy of Medical Sciences, Taian, People’s Republic of China; b School of Public Health, Shandong First Medical University & Shandong Academy of Medical Sciences, Taian, People’s Republic of China; c National Virus Reference Laboratory, School of Medicine, University College Dublin, Dublin, Ireland; d Global Station for Zoonosis Control, Global Institution for Collaborative Research and Education (GI-CoRE), Hokkaido University, Sapporo, Japan; e Department of Obstetrics and Gynecology, Central Hospital of Taian, Taian, People’s Republic of China

**Keywords:** HFMD, coxsackievirus A4, murine model, inactivated vaccine, antiviral drugs

## Abstract

Coxsackievirus A4 (CVA4) infection can cause hand, foot and mouth disease (HFMD), an epidemic illness affecting neonatal and paediatric cohorts, which can develop to severe neurological disease with high mortality. In this study, we established the first ICR mouse model of CVA4 infection for the evaluation of inactivated vaccines and antiviral drug screening. The CVA4 YT226R strain was selected to infect the neonatal mice and three infectious factors were optimized to establish the infection model. The 3-day-old neonatal mice exhibited clinical symptoms such as hind limb paralysis and death. The severe inflammatory reactions were closely related to the abnormal expression of the acute phase response proinflammatory cytokine IL-6 and an imbalance in the IFN-γ/IL-4 ratio. Importantly, the inactivated CVA4 whole-virus vaccine induced humoral immune responses in adult females and the maternal antibodies afforded mice complete protection against lethal dose challenges of homologous or heterologous CVA4 strains. Both IFN-α2a and antiserum inhibited the replication of CVA4 and increased the survival rates of neonatal mice during the early stages of infection. This neonatal murine model of CVA4 infection will be useful for the development of prophylactic and therapeutic vaccines and for screening of antiviral drugs targeting CVA4 to decrease morbidity and mortality.

## Introduction

Coxsackievirus A4 (CVA4) belongs to the family *Picornaviridae*, genus *Enterovirus* of linear, non-enveloped, positive polarity single-stranded RNA viruses, which principally cause herpangina and hand, foot and mouth disease (HFMD) in children [1]. Prior to 2009, viral pathogens associated with HFMD included enterovirus A71 (EVA71) and CVA16; however, in recent years, other types of enteroviruses (EVs), such as CVA4, CVA6 and CVA10 have increased in prevalence [2–3]. Numerous outbreaks of CVA4 have occurred during recent years in different regions in China, such as Fujian [4], Shandong [5, 6], Sichuan [7], Yunnan [8], and Jiangsu [9]. Other countries, such as Thailand [2, 10], Australia [11] and Italy [12] have also reported CVA4 infections. Genetic recombination between CVA4 and other EVs has been described generating novel emerging strains which may be associated with more severe disease [13]. Although the majority of CVA4 and EV infections are usually subclinical, these agents are highly contagious and exhibit a broad spectrum of disease presentation in symptomatic cases ranging from mild symptoms such as erythematous maculopapular exanthema [12] to severe outcomes such as aseptic meningitis, myocarditis and acute flaccid paralysis [14, 15]. Given the increased frequency of CVA4 circulation globally [11, 16, 17], evidence for complex spatiotemporal dynamics in Asia-Europe [18] and the emergence of novel CVA recombinants with the potential to spread rapidly in immunologically naïve populations [13], greater focus is required both on surveillance and also the development of prophylactic and therapeutic approaches.

At present, there are no specific drugs for the clinical treatment of HFMD. HFMD is currently treated with broad-spectrum antiviral drugs, such as ribavirin [19, 20] and glucocorticoids [21, 22], which can cause damage to the innate immune system [23] and lead to haematopoietic system disorders [24]. Prophylactic approaches employing vaccination to prevent EV transmission now exist [25] and are being combined with existing non-EV vaccines to simplify childhood vaccination regimes [26]. Antiviral research on EVs is lacking and the establishment of CVA4 infection models for drug development and the examination of the immunogenicity of inactivated CVA4 vaccines is urgently needed. With the wide application of the inactivated EVA71 vaccine among children under five in China [27, 28], it is conceivable that the EV-associated HFMD pathogens will alter in the future as the immune protection can last for >5 years [29]. Incidence rates of other EVs will likely increase gradually, therefore it is imperative to study the pathogenic mechanisms of other EVs, such as CVA4, and to develop antiviral drugs and vaccines accordingly. In the present study, a CVA4 neonatal murine model was established to analyse viral tissue tropism and pathology and to better elucidate the pathogenicity associated with CVA4 infection. Employing this infection model, we have evaluated the antiviral effects of immune sera and the protective effects of cytokines and maternal antibodies against CVA4.

## Materials and methods

### Ethics statement

The experimental animals were outbred SPF ICR mice [qualification number: SCXK (Beijing) 2012-0001]. The feeding and processing of the mouse were subject to the animal welfare and ethical guidance of the Shandong Provincial Laboratory Animal Management Committee.

### Cells and CVA4 viral strain

Human rhabdomyosarcoma (RD) cells were kindly provided by the Shandong Center for Disease Control and Prevention. RD cells were cultured in minimum essential medium (MEM) containing 10% fetal bovine serum (Gibco, Invitrogen, USA). The RD cells inoculated with the CVA4 YT226R strain showed obvious cytopathic effects (CPE) after 24 h. The phylogenetic analysis showed that the strain belonged to the D2 genotype (Figure S1). After the virus supernatant was centrifuged and filtered through a 0.22 μm filter (Pall Corporation, Germany), CVA4 titre was quantified by the Reed-Muench method [30] to be 3.98 × 10^8^ TCID_50_/mL.

### Establishment of the neonatal murine model of CVA4 infection

In order to establish the CVA4 infection model, the optimal challenge dose was first experimentally determined, and the 3-day-old neonatal mice were inoculated with the YT226R strain prepared by a serial 10-fold dilutions with MEM (10^7.0^-10^3.0^ TCID_50_/mouse). In order to obtain the optimal age of the mice for infection, 1-, 3-, 7- and 9-day-old neonatal mice were selected, and 10^4.0^ TCID_50_/mouse of YT226R was inoculated via the intramuscular (i.m.) route. In order to obtain the optimal infection route, 3-day-old neonatal mice were inoculated with 10^4.0^ TCID_50_/mouse YT226R via the i.m., intraperitoneal (i.p.) or intracoelomic (i.c.) routes, respectively. Three-day-old neonatal mice in the negative control group were inoculated with the same volume of MEM via the i.m. route. After the neonatal mice were infected, the body-weight changes, clinical scores, and survival rates were observed and recorded daily. The clinical scores were based on the following criteria[31]: 0, healthy; 1, lethargy or listlessness; 2, wasting or hind limb weakness; 3, single hind limb paralysis; 4, double hind limb paralysis; 5, death.

### Histopathologic and immunohistochemical staining

The 3-day-old neonatal mice were i.m. inoculated with 100 LD_50_ of CVA4 YT226R strain (10^4.0^ TCID_50_/mouse) and mice with a clinical score of ≥4 were anaesthetized with ether and sacrificed. The lungs, hind limb muscles, spinal muscles and heart were separated, fixed, dehydrated, permeabilized and embedded in paraffin, which was then sliced into 4 μm sections. After staining with haematoxylin and eosin, histopathological analysis was performed.

Immunohistochemistry was performed using an avidin–biotin–immunoperoxidase technique. Briefly, tissue sections were dewaxed, dehydrated, and microwaved for 20 min at 99°C in a citrate buffer. Polyclonal mouse anti-CVA10 antibody (1:100 dilution; Genecreate, Wuhan, China) was applied for 2 h at 37°C. A secondary biotinylated goat anti-mouse immunoglobulin G (1:1000 dilution; Beyotime, Shanghai, China) antibody was added, followed by avidin–biotin–peroxidase complex and 3,3′-diaminobenzidine tetrahydrochloride chromogen. Tissues were counterstained with haematoxylin.

### Dynamic monitoring of CVA4 viral loads in different organs

The 3-day-old neonatal mice were inoculated with 100 LD_50_ of YT226R. Between 1 and 5 days post infection (dpi), following euthanization, the lungs, hind limb muscles, spine muscles, blood, intestine, brain and heart tissues of the mice were collected every 24 h. Total RNA was extracted from individual tissues of infected mice with TRIzol reagent (TaKaRa, Dalian, China) and reverse transcribed with random hexamers and Moloney murine leukaemia virus reverse transcriptase (TaKaRa, Dalian, China) to generate cDNA in accordance with the manufacturer's instructions. The CVA4 load was detected by qRT-PCR using the primers 5′-GGCCTCACTCAGAGACATCTAACC-3′ and 5′-GTCTAGGGACCCATGCCCTCACT-3′. The fluorescent probe was FAM-TGGACAGCCCACCACCGCAAGTGT-BHQ1. The qRT-PCR reaction conditions were set up based on a previously published detection method for EVs [32]. A standard curve was established: *Y*=−3.2995*X* + 30.96, *R*^2 ^= 0.99, and the detection limit was 3.96 copies/µL.

### Cytokine detection

The 3-day-old neonatal mice were challenged with 100 LD_50_ of YT226R. Between 1 and 7 dpi, peripheral blood (PB) was collected from the infected group and the negative-control group randomly selected from the neonatal mice after anaesthesia. The serum was separated after PB coagulation. The concentration of IFN-γ, IL-4, IL-6, IL-10 and TNF-α in the serum was determined with individual mouse ELISA detection kits (Multisciences Biotechnology Co., Ltd., Hangzhou, China). Briefly, the serum was mixed with diluted antibody and then horseradish peroxidase-labeled streptavidin and the chromogenic substrate TMB were added sequentially, according to the manufacturer's instructions. Optical density values at 450 and 630 nm were measured using a microplate reader and the cytokine concentration calculated by a linear equation. The theoretical limit of detection is 1.74 pg/mL (IFN-γ), 0.22 pg/mL (IL-4); 1.17 pg/mL (IL-6), 1.17 pg/mL (IL-10) and 1.63 pg/mL (TNF-α).

### Anti-viral effects of ribavirin and cytokines *in vitro* and *in vivo*

RD cells with a confluency of 80-90% in 96-well plates were inoculated with 10^2.0^ TCID_50_ YT226R for 1 h. Ribavirin (20 μg) and IL-1β (1250 U), IL-6 (12.5 U), IFN-λ1 (125 ng), IFN-γ (25 U) and IFN-α2a (125 U) were added to the cells. CPE was observed at 16, 24, 32, 40 and 48 h after viral inoculation. The inhibition rate of CVA4 replication between 8 and 48 h post infection (hpi) was detected with the CCK8 kit (Solarbio Science & Technology Co., Ltd., Beijing, China). The viral load was determined by qRT-PCR.

The 3-day-old neonatal mice were i.m. challenged with 100 LD_50_ of YT226R. Ribavirin (100 μg), IL-1β (2000 U), IL-6 (1000 U), IFN-λ1 (1125 U), IFN-γ (833.25 U) and IFN-α2a (666.75 U) were injected 1 h after infection. At 1 dpi, the same amount of ribavirin and cytokines were re-injected. The body weights, clinical scores and survival rates of the neonatal mice were recorded daily until 13 dpi.

### Cross-immunity of CVA4 antiserum

YT226R (3.98 × 10^8^ TCID_50_/mL) was inactivated by adding 37% formaldehyde (Sinopharm Group, Beijing, China) to viral suspensions to a final concentration of 250 μg/mL for 5 d at 37°C before mixing (1:1) with aluminium hydroxide gel (InvivoGen, San Diego, CA, USA) to prepare the inactivated CVA4 whole-virus vaccine. A total of 150 µL of inactivated CVA4 vaccine (corresponding to 7.9 × 10^4^ TCID_50_/mouse) was inoculated into 6-week-old adult mice (randomly selected), boosted once after two weeks, and the serum was collected after another two weeks. The CVA4 antiserum was heat-inactivated at 56°C for 30 min. The antibody titre of the antiserum was determined by microneutralization assay (geometric mean titre [GMT], 3444.32). The RD cells were placed in a 96-well plate (5000 cells/well) and cultured for 24 h. The antiserum was diluted 512-fold with MEM, and then added to RD cells, which were incubated for 2 h, before 100 TCID_50_ of CVA4, EVA71, CVA16 and CVA6 were added (MOI = 0.01), respectively. The supernatant was collected at 24, 48, and 72 hpi, respectively, 10-fold diluted and inoculated to RD cells in 96-well plates. The RD cells were observed for development of CPE, and the virus titre of each experimental group was calculated by the Reed-Muench method[30] after considering the dilution factor.

### Maternal antibody protection

Six-week-old adult female mice (*n *= 2 per group) were subcutaneously injected with 150 μL of formaldehyde-inactivated whole virus CVA4. After the female was pregnant, the booster comprised the same dose of the vaccine at a 2-week interval. After delivery, the 3-day-old pups were injected with 100 LD_50_ of the homologous CVA4 YT226R strain or 100 LD_50_ of heterologous CVA4 strains (LY124R/SDLY/CVA4/China/2014; H337/SDHZ/CVA4/China/2015; LC16114/SDLC/CVA4/China/2016) or 100 LD_50_ of other EVs: CVA6 (WF057R/CVA6/China/2014), CVA16 (TA271/CVA16/China/2015) and EVA71 (SD004R/EVA71/China/2015). The body weights, clinical scores and survival rates of the neonatal mice were measured daily.

### Therapeutic effect of anti-CVA4 serum

The 3-day-old neonatal mice were infected with 100 LD_50_ of YT226R. After infection, the mice with clinical scores of 1–2 were selected for early treatment and the neonatal mice with clinical scores ≥3 were selected for late treatment. The CVA4 antiserum (GMT = 3444.32) was mixed with MEM at a ratio of 1:1, 1:10, 1:100 and 1:1000, respectively. The neonatal mice in each group were i.m. injected with 50 μL of serum. The antisera were injected again after 48 h. The body weights, clinical scores and survival rates of the neonatal mice in each group were recorded daily until 12 days post treatment (dpt). A 50% passive immunoprotective dose (ED_50_) of the CVA4 antiserum was calculated.

### Statistical analyses

Statistical analyses were performed using the GraphPad Prism 5 software (GraphPad 5 Software, San Diego, CA, USA). Survival and mortality of the neonatal mice in each group were evaluated using the Mantel–Cox log rank software. Tissue viral loads, cytokine levels, TCID_50_, LD_50_ and ED_50_ validation were performed using a two-tailed Mann–Whitney U test. Quantitative variables were analysed by means, *t*-test or variance analysis. *P *< .05 indicates significant difference.

## Accession number(s)

The genome sequences of the clinical isolates characterized in the present study, YT226R/SDYT/CVA4/China/2015, LY124R/SDLY/CVA4/China/2014, H337/SDHZ/CVA4/China/2015, LC16114/SDLC/CVA4/China/2016, WF057R/CVA6/China/2014, TA271/CVA16/China/2015 and SD004R/EVA71/China/2015 have been deposited in GenBank under accession numbers MH086040, MH086031, MH086030, MH086049, KX752785, MG674827 and KY315729.

## Results

### Establishment of the CVA4 infection model

**Figure 1.**
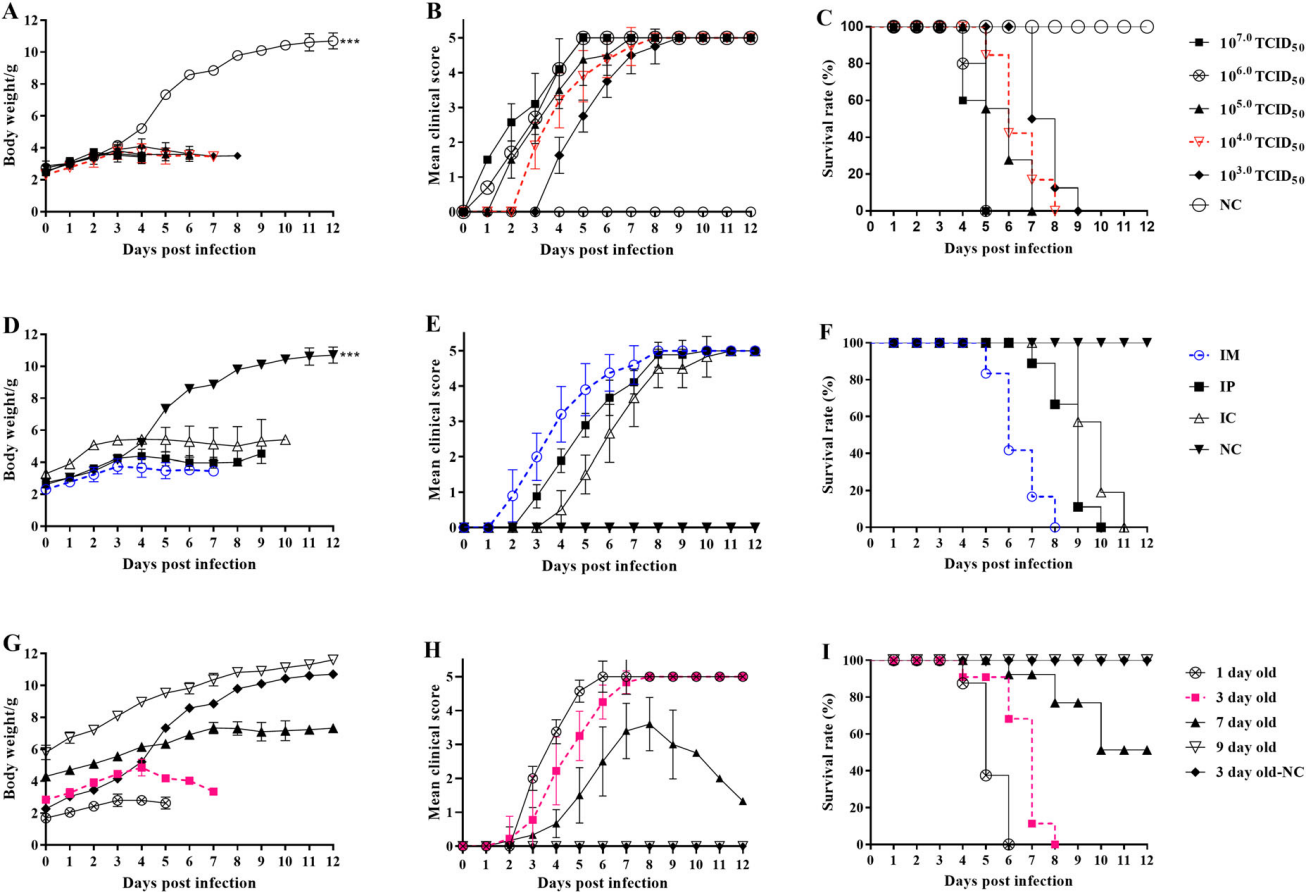
Establishment of the CVA4 infection model. The CVA4 infection ICR mouse model was established by optimizing three factors: challenge dose, challenge route and mouse age. The body weights (A, B, and C), clinical scores (D, E and F), and survival rates (G, H and I) in each group of neonatal mice (*n *= 10–15) were measured. The LD_50_ was calculated as 10^2.0^ TCID_50_/mouse. *P < .05; *** * < 0.01; ***P < .001; n.s. non-significant result.

In order to optimize the challenge dose, the 3-day-old neonatal mice were administered 10-fold serial dilutions (10^7.0^-10^3.0^ TCID_50_/ml) of the CVA4 strain YT226R (D2 genotype, see Figure S1 in the supplemental material) via the i.m. route. The group with challenge doses ≥10^5.0^ TCID_50_/mouse began to exhibit clinical symptoms ≤2 dpi, while the group with a challenge dose of 10^4.0^ TCID_50_/mouse began to show symptoms at 3–4 dpi and all animals died by 8 dpi (Figure 1(A–C)). Compared with the other groups, the group with the challenge dose of 10^4.0^ TCID_50_/mouse had milder clinical symptoms with a longer duration of illness. The symptoms included a moribund state, ataxia, nerve and single or double hind limb paralysis. Hence, the titre of 10^4.0^ TCID_50_/mouse was chosen as the optimal challenge dose. The 3-day-old neonatal mice were next challenged with 10^4.0^ TCID_50_/mouse via the i.m., i.p. and i.c. routes, respectively. The mice in the i.m. group began to exhibit neurological symptoms at 3 dpi and all died by 8 dpi. The mice in both the i.p. and i.c. groups died ≥10 dpi (Figure 1(D–F)). Hence, the i.m. route was chosen as the neonatal mice were more susceptible to CVA4 via this route. In order to optimize the age of the neonatal mice, 1-, 3-, 7- and 9-day-old neonatal mice were challenged with a dose of 10^4.0^ TCID_50_/mouse via the i.m. route. All 3-day-old neonatal mice died by 8 dpi, while the 7- and 9-day-old neonatal mice had mortality rates ≤50% at 12 dpi with clinical scores ≤3 (Figure 1(G–I)). Furthermore, due to the large variance and uneven distribution of the data for the 1-day-old neonatal mice, the 3-day-old neonatal mice were selected for the infection model.

### Pathology and immunohistochemistry

**Figure 2.**
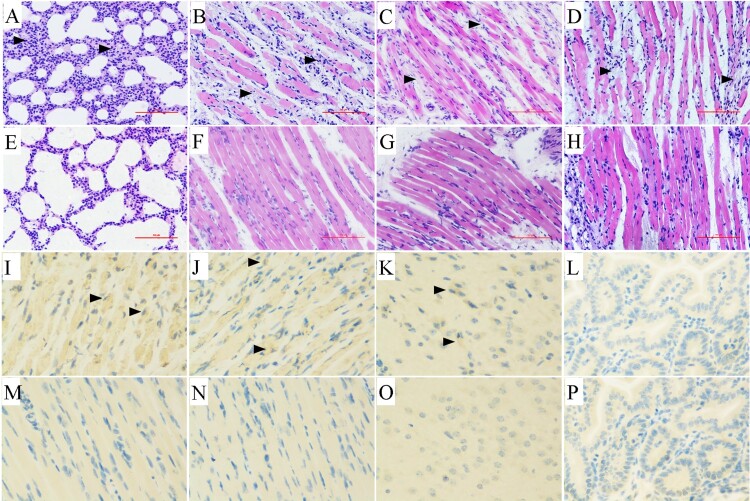
Histopathological and immunohistochemical examination (200×). After the 3-day-old neonatal mice were i.m. challenged with a lethal dose of YT226R. Tissue samples from the lungs (A), hind limb muscle (B), spinal muscle (C) and heart (D) were subjected to pathology analysis. No obvious histological changes (E–H) were observed in each tissue of the negative control group. The IHC analysis indicated that the viral antigen was differentially distributed in affected tissues: hind limb muscle (I), spine muscle (J) and brain (K), but not in intestine (L). Results for noninfected mice were employed as a negative control (M–P).

The 3-day-old neonatal mice were challenged with a lethal dose of the CVA4 YT226R strain (100 LD_50_) via i.m. administration. When the clinical scores of the neonatal mice reached 4, the animals were euthanized and the tissues were surgically removed, sectioned and HE staining was performed. Pathological examination showed a significantly widened alveolar space, vasodilatation, hyperaemia, and interstitial oedema with large numbers of a predominantly monocyte-based inflammatory cell infiltrate (Figure 2(A)). The hind limb muscles and spinal muscles were severely affected, and both showed muscle fibre necrosis and muscle bundle rupture (Figure 2(B,C)). In the late stage of infection, large numbers of a diffuse lymphocytic and mononuclear cell infiltrate were evident and associated with myocardial rupture (Figure 2(D)). There were no obvious pathological changes in the tissues of the negative control group (Figure 2(E–H)), indicating that CVA4 infection can cause severe pathology, tissue lesions and inflammatory reactions. In the Immunohistochemistry (IHC) experiments, the viral antigen of CVA4 was detected in hind limb muscle (Figure 2(I)), spine muscle (Figure 2(J)) and brain (Figure. 2(K)) of neonatal mice, but not in intestine (Figure 2(L)), which may be related to the route of infection. There was no antigen detected in the negative control groups (Figure 2(M–P)).

### Dynamic monitoring of viral loads in different organs

**Figure 3.**
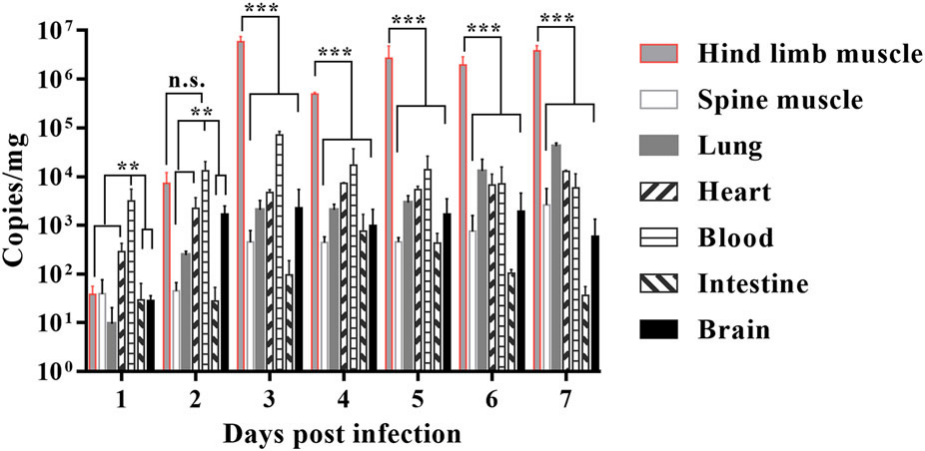
Changes in the CVA4 viral load in tissues. The 3-day-old neonatal mice were challenged with a lethal dose of CVA4 strain YT226R and the viral loads in the hind limb muscle, spine muscle, lung, heart, blood, intestine and brain tissues were determined by qRT-PCR (*n *= 3).

In order to monitor the changes of CVA4 viral loads in different organs by qRT-PCR, the lungs, hind limb muscles, spine muscles, blood, intestine, brain and heart tissues were separated between 1 and 5 dpi after the mice were i.m. administered with a lethal dose of YT226R. The results showed that the viral copy number in each organ was low at 1 dpi. The viral load in the blood was the highest, leading to viremia. At later time points, the virus spread and replicated rapidly, and the copy number peaked and stabilized at 3 dpi (Figure 3). Among the organs, the hind limb muscle had the highest viral loads with copy numbers of 10^6^ copies/mg from 3 to 5 dpi, which was >2–3 log_10_ greater than those in other organs over the same period. CVA4 in the intestine showed a relatively low level of replication (27.267–429.256 copies/mg). The CVA4 copy number in the brain increased significantly after 2 dpi, reaching 1167.851 copies/mg, showing a high level of replication during 2–7 dpi. The results indicate that CVA4 has a pronounced tropism for muscle tissue and the rapid replication of the virus in the hind limb musculature causes rupture and hind limb paralysis.

### Expression level of inflammatory cytokines

**Figure 4.**
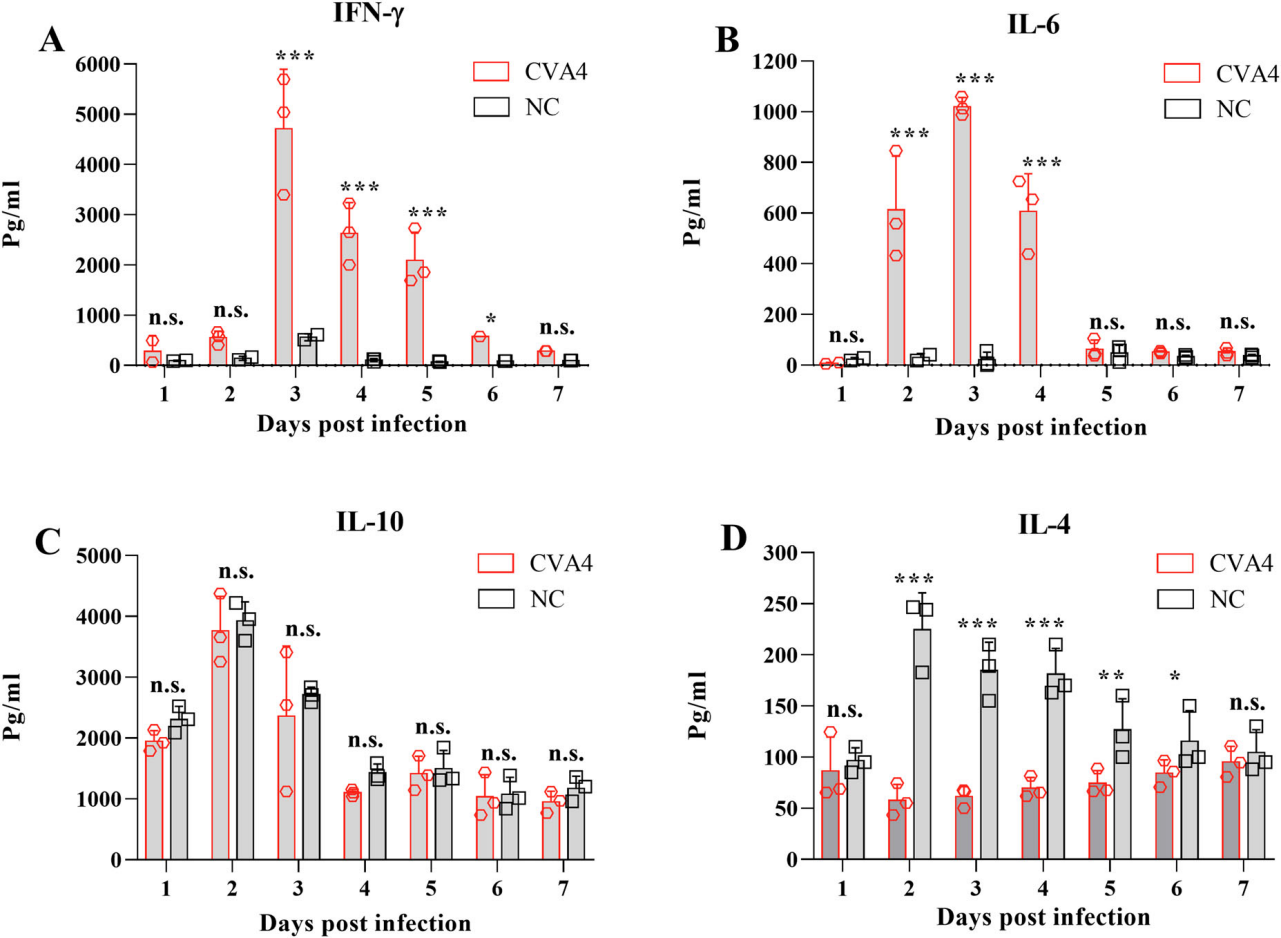
Cytokine expression in the CVA4 infection model. After the 3-day-old neonatal mice were inoculated with a lethal dose of YT226R, the expression levels of IFN-γ (A), IL-6 (B), IL-10 (C), and IL-4 (D) were detected by ELISA from PB of the neonatal mice at 1–5 dpi (*n *= 3). NC: negative control.

In order to detect the changes in expression of cytokines during CVA4 infection, cytokines in PB of the neonatal mice at different time points (1–7 dpi) were detected by ELISA. The results showed that the expression levels of IFN-γ and IL-6 were low in the early stages of infection (1 dpi), and then increased to peaks of 4800 and 1020 pg/mL, respectively, by 3 dpi which were significantly higher than those of the negative control group (Figure 4(A,B)). The expression level of IFN-γ and IL-6 gradually decreased from 3 dpi and reached 289 and 52 pg/mL at 7 dpi, respectively. There were no significant changes in the expression of IL-10 in the infected neonatal mice between 1 and 7 dpi compared with those of the negative control group (*P *= .548, Figure 4(C)), while the expression of IL-4 was significantly lower than that of the negative control group from 2 to 7 dpi (Figure 4(D)). Therefore, abnormal expression of IFN-γ and IL-4 led to an imbalance of the IFN-γ/IL-4 ratio.

### In vitro and in vivo antiviral effects of ribavirin and cytokines

**Figure 5.**
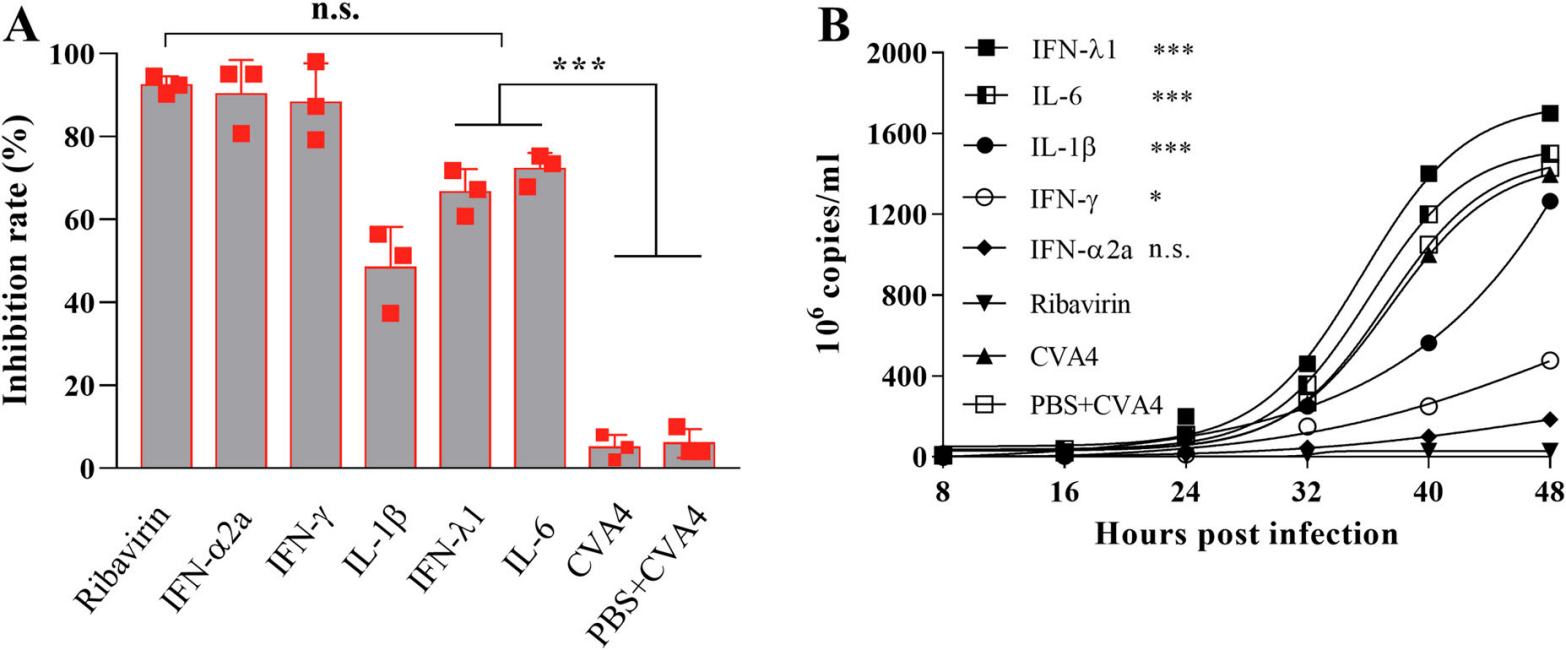
Anti-CVA4 effects of ribavirin and cytokines *in vitro*. One hour after the RD cells were challenged with YT226R, ribavirin and different cytokines were added, respectively. CCK8 and qRT-PCR were employed to determine the inhibition rates of ribavirin and different cytokines on CVA4 replication (A), and the viral loads between 8 and 48 hpi (B), respectively.

RD cells were first i.m. inoculated with 10^2.0^ TCID_50_ of YT226R at an MOI of 0.01, and then treated with ribavirin and a cytokine panel 48 hpi. The maximum inhibition rates of ribavirin and different cytokines on CVA4 replication were detected by the CCK8 method. The inhibition rates of ribavirin, IFN-α2a, IFN-γ, IL-1β, IFN-λ1 and IL-6 were found to be 93.03%, 92.37%, 91.03%, 48.38%, 66.60% and 72.19% (Figure 5(A)), respectively. At the same time, the viral loads in the supernatants from each group were determined, which demonstrated that the viral copy numbers in the cells treated with IFN-λ1, IL-6 and IL-1β were significantly higher than those in the cells treated by ribavirin, IFN-α2a and IFN-γ (Figure 5(B)). These results indicated that IFN-γ and particularly IFN-α2a had comparable anti-CVA4 effects compared with ribavirin *in vitro*.

**Figure 6.**
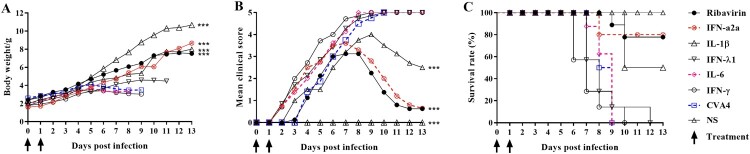
Anti-CVA4 effects of ribavirin and cytokines *in vivo*. One hour after the 3-day-old neonatal mice (*n *= 10–15) were challenged with YT226R, ribavirin and different cytokines were administered for the first time. Ribavirin and the cytokines were re-administered at 24 hpi. The body weights (A) and clinical scores (B) and survival rates (C) of the neonatal mice were measured.

Neonatal mice were challenged with a lethal dose of YT226R, and therapeutic doses of ribavirin, IFN-α2a and IL-1β were injected twice (1 h and 2 dpi) via the i.m. route. By 13 dpi, the survival rates of the mice were 80%, 82%, 50%, and the clinical scores were 1.25, 1.47, and 2.50, respectively, and the body weight showed a significant upward trend for the groups treated with ribavirin, IFN-α2a, and IL-1β (Figure 6). The mice treated by IFN-γ, IFN-λ1 and IL-6 showed severe clinical symptoms and all died before 13 dpi. Therefore, compared with ribavirin, IFN-α2a could also inhibit the replication of CVA4 *in vivo* and improve the survival rate of the neonatal mice.

### Cross-protection of CVA4 antiserum

**Figure 7.**
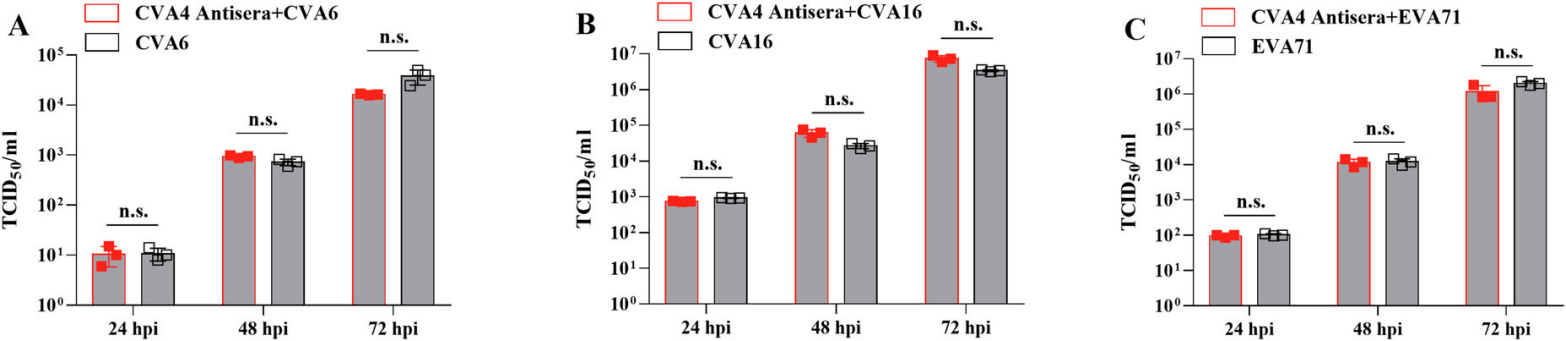
Cross-protection of the anti-CVA4 serum. CVA4 antiserum was co-incubated with CVA4, CVA6, CVA16, and EVA71 in RD cells for 24, 48 and 72 hpi, respectively, and the viral titre in the supernatants were determined by assessing development of CPE (*n *= 3).

In order to test whether anti-CVA4 antiserum can neutralize CVA4, CVA6, CVA16 and EVA71 infection, a 512-fold dilution of antiserum (GMT = 3444.32) was incubated with these EVs following inoculation in RD cells for 24, 48, and 72 h, respectively and the viral titre in cell supernatants was determined by assessment of CPE. Antiserum was found to significantly neutralize the infectivity of CVA4: CPE was not observed at 24, 48 and 72 h after CVA4 was co-incubated with antiserum. However, the CVA4 antiserum did not neutralize CVA6, CVA16 and EVA71 (Figure 7). Therefore, CVA4 antiserum did not have a cross-immunological protective effect against other EV types *in vitro*.

### Maternal antibody protection

**Figure 8.**
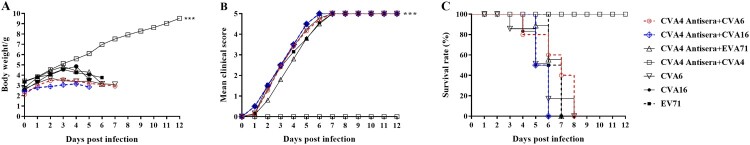
Protective effect of CVA4 maternal antibodies. The 6-week-old adult females were immunized twice with the inactivated CVA4 vaccine. After delivery, the pups (*n *= 10–15) were inoculated with a lethal dose of homologous or heterologous CVA4, CVA6, CVA16 and EVA71 strains on postnatal day 3.

Six-week-old adult females were immunized twice with the inactivated CVA4 whole-virus vaccine supplemented with an aluminium hydroxide adjuvant. After delivery, the pups were administered with lethal doses of homologous or heterologous CVA4 strains and other EVs: CVA6, CVA16 and EVA71 on postnatal day 3, respectively. It was found that the weight gain of the YT226R (CVA4) group was normal during the observation period without obvious symptoms. The survival rate was 100%, and the maternal antibody could prevent the infection of the CVA4 heterologous strains, including H337, LY124R, and LC16114 (see Figure S2 in the supplemental material). However, the symptoms of the CVA16, EVA71, and CVA6-infected mice were severe, and all mice died by 6, 7, and 8 dpi, respectively. The weight gain in the CVA16, EVA71, and CVA6 treated groups was significantly decreased (Figure 8). In summary, the CVA4 vaccine could induce humoral immune responses in adult females, and the maternal antibody could provide 100% protection against homologous and heterologous CVA4 challenge; however, there was no cross-protective effects elicited by the CVA4 immune response to afford protection to CVA16, EVA71 and CVA6 *in viv*o.

### Therapeutic effect of anti-CVA4 serum

**Figure 9.**
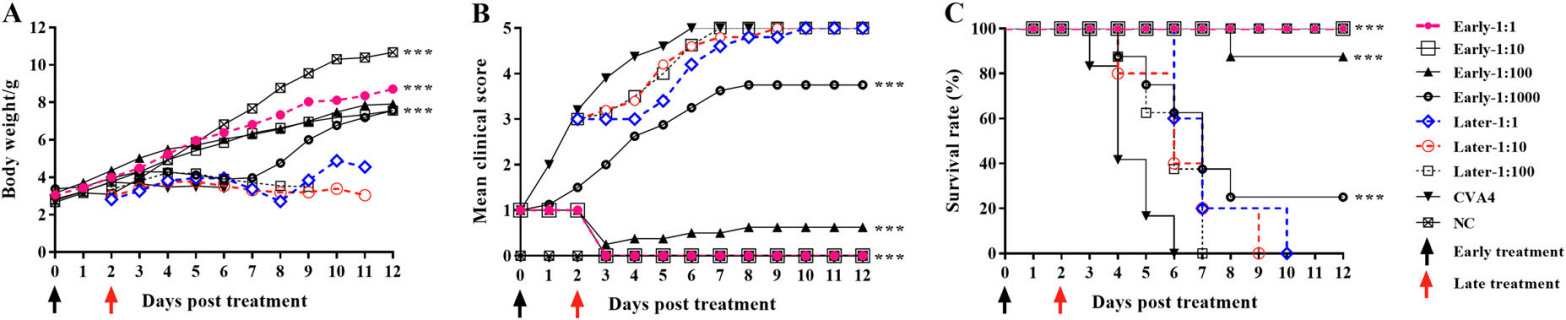
Protective effects of the anti-CVA4 serum. The 3-day-old neonatal mice (*n *= 10–15) were challenged with a lethal dose of YT226R. The neonatal mice with a clinical score of 1–2 were treated with the antisera diluted at a ratio of 1:1, 1:10, 1:100 and 1:1000 twice (Early stage treatment). Also, the neonatal mice with a clinical score of ≥3 were treated with the antisera twice (Late stage treatment).

After challenge with a lethal dose of YT226R, 3-day-old neonatal mice with a clinical score of ≤2 were twice injected with CVA4 antiserum treatment (GMT = 3444.32) with dilution ratios of 1:1, 1:10, 1:100, and 1:1000, respectively. At 12 dpt, the clinical scores were 0, 0, 0.63, and 3.75, and the survival rates were 100%, 100%, 85%, and 25%, respectively (Figure 9), and the calculated ED_50_ was 199.05. Although the clinical symptoms of the neonatal mice were delayed after late treatment of antiserum (clinical score ≥3), the mice treated with 1:1, 1:10, and 1:100 dilutions of sera died by 10, 9 and 7 dpt, respectively. The results suggested that antibody treatment should be administered in the early stages of viral infection to mitigate development of severe disease.

## Discussion

The infection model provides a foundation for the evaluation of the efficacy of novel antiviral and vaccine candidates. EVA71 vaccines have been successfully developed employing mouse [33, 34] and primate [35, 36] models. Similarly, based on animal models, CVA16, CVA6 and CVA10 vaccines are currently under development [31, 37, 38]. Due to the significant increase in the incidence of CVA4-associated HFMD infections worldwide in recent years [2, 9, 39], it is urgently required to establish a CVA4 infection model for evaluation of both inactivated and attenuated live CVA4 vaccines and for the screening of existing and novel antiviral drugs. In this study, we found inoculation of CVA4 by the i.m. route caused the most rapid onset of illness in the neonatal ICR mice, which we hypothesize may be attributable to the pronounced tropism and elevated replication of CVA4 in muscle tissue and the subsequent systemic dissemination of the virus through the circulatory system. Neonatal mice (≤ 3 d) were most susceptible to CVA4 and showed high mortality rates. By optimizing these factors, a CVA4 infection model with high levels of repeatability for clinical scores, disease onset and duration of illness was established.

In the present study, we found that CVA4 had a copy number of up to 10^6^ copies/mg tissue in the musculature, which was associated with rupture and necrosis of muscle fibres and muscle bundles. Despite the relatively low viral load in the lungs (10^3^ copies/mg), the mice showed severe diffuse alveolar damage and moderate inflammatory infiltration. EV infections can induce the expression of proinflammatory cytokines as part of the innate immune response and immune regulation to eliminate viruses. However, it is thought that the overwhelming viral replication combined with increased levels of cytokines is one of the key factors inducing severe HFMD [40]. Indeed, high levels of cytokines and chemokines in cerebrospinal fluid, including TNF-α, IL-6, IL-10, IL-13, and IFN-γ, are associated with brain stem encephalitis and pulmonary oedema caused by EVA71 infection [41–43.] In this study, the expression levels of the proinflammatory cytokines IL-6 and IFN-γ were abnormally high after the lethal dose of CVA4 was administered to neonatal mice. This phenomenon is similar to that observed in children infected with EVA71, in which the expression of IL-6 and IFN-γ in serum was significantly elevated [44]. Some researchers have proposed that sustained high levels of IL-6 alone can cause severe tissue damage. In support of this, anti-IL-6 treatments significantly reduce tissue damage and enhance immune cell activity [45]. Similarly, we found that mice challenged with CVA4 had higher clinical scores after treatment with exogenous IL-6 which accelerated the death of the neonatal mice. In addition, after EV infection in human neuronal cells, the expression levels of IFN-γ secreted by Th1 cells is increased, inhibiting the secretion of IL-4 by Th2 cells and resulting in an imbalance of the IFN-γ/IL-4 ratio. While the infected cells were rapidly cleared, contemporaneously, excessive inflammatory reactions and cellular immune responses were also produced [46, 47]. The expression level of IL-4 was significantly lower in the CVA4-infected mice than that in the control group (mean value: 73.5 vs 165.4 pg/mL) in this study. Therefore, we consider that the CVA4-induced high levels of IL-6 and an imbalance of the IFN-γ/IL-4 ratio are likely correlated with severe pathological damages.

Ribavirin is a hypoxanthine nucleoside analog which acts as a broad-spectrum antiviral drug inducing lethal mutations during viral RNA replication [48]. Ribavirin inhibits EVA71 replication and reduces paralysis and mortality rates in EVA71-infected mice [20]. It has been clinically applied in the treatment of HFMD and has anti-CVA4 effect *in vitro* and *in vivo*; however, these nucleoside drugs have side effects with long-term administration leading to haematopoietic dysfunction and allergic reactions in children [23]. Surprisingly, IFN-α2a had strong anti-CVA4 effects (comparable to ribavirin), which significantly reduced the copy number of CVA4. Our *in vivo* experiments showed that IFN-α2a could inhibit CVA4 replication and increase the survival rates of neonatal mice. It is important to note that the protective effect of cytokines is closely related to the time of administration [49]. The antiviral effects of cytokines (such as IFN-γ and IL-6) mainly functioned in the early stage of viral infection and when administered in the later stages, the cytokines did not alleviate symptoms but instead, led to further deterioration of the disease, which may be due to aggravated immunopathology [50].

With the increasing incidence of CVA4, inoculation with an inactivated CVA4 vaccine may be the most effective measure to prevent infections. A previous study found that human antibodies against EVA71 and CVA16 infections were detected in the serum after 35 weeks of pregnancy [51]. The inactivated CVA4 vaccine developed in this study also induced protective antibodies in adult female mice and could be passed on to offspring to protect the pups from the lethal dose of CVA4 challenge. Importantly, the protection rate of pups challenged with both homologous and heterologous CVA4 strains was 100%. However, it did not have neutralizing ability against CVA16, EVA71 and CVA6 strains. Therefore, developing multivalent vaccines using different antigens which elicit balanced immunological responses and afford equivalent protection to each target are urgently needed.

Anti-CVA4 serum could alleviate the early clinical symptoms of the neonatal mice and the surviving animals fully recovered. However, it had no therapeutic effect on late-stage infected neonatal mice, which may be because the late illness onset of the neonatal mice (≥3 dpi) had irreversible systemic inflammatory responses and/or established nervous system damage. Similarly, severe cases of HFMD caused by EVA71 infection usually progress rapidly. Although the proportion of critically ill patients with EVs infection is ≤1.1%, the number of annual cases exceeds one million in China [52]. Therefore, patients with HFMD should be treated with antiviral and symptomatic treatments in a timely manner before irreversible disease deterioration.

In summary, we have established the first stable neonatal mice model of CVA4 infection which displayed consistent pathological changes and demonstrated the immunogenicity of an inactivated CVA4 whole-virus vaccine. IFN-α2a and antiserum had effective anti-CVA4 effects *in vitro* and *in vivo*. This neonatal murine model lays a foundation for studying the pathogenesis of CVA4 and for evaluating the effectiveness of vaccines and screening of antiviral drugs.

## Supplementary Material

Supplemental Material
