# Supplementary material for: A neonatal murine model of coxsackievirus A4 infection for evaluation of vaccines and antiviral drugs
Source: Emerg Microbes Infect. 2019 Oct 9;8(1):1445–55. doi: 10.1080/22221751.2019.1673135 (PMC6792045; doi:10.1080/22221751.2019.1673135)
Supplement: Supplemental Material [file TEMI_A_1673135_SM2939.zip › Supplementary_Figs..docx]

**Fig. S1 Phylogenetic tree based on the alignment of the complete genome sequence of international CVA4 strains (7.4 kb).** The neighbor-joining method was used to construct the phylogenetic tree and was determined for 1000 replicates with random seeds. Only strong bootstrap values (>70%) are shown.

Indicates the CVA4 YT226R strain used in the present study to establish the neonatal mouse model of CVA4 infection.

Indicates three clinical isolates of CVA4 from different regions of Shandong: LY124R, H337 and LC16114.

**Fig. S2 Maternal immunization with inactivated CVA4 vaccine protects pups against lethal challenge doses of various CVA4 strains.** After delivery, the pups were i.m. challenged with lethal doses (100 LD_50_) with the following CVA4 strains: YT226R, LY124R, H337, and LC16114 on postnatal day 3. The body weights (**A**), clinical symptoms (**B**), and survival rates (**C**) of the challenged pups were monitored until 12 dpi.
