# Supplementary figures and images for: A neonatal murine model of coxsackievirus A4 infection for evaluation of vaccines and antiviral drugs
Source: Emerg Microbes Infect. 2019 Oct 9;8(1):1445–55. doi: 10.1080/22221751.2019.1673135 (PMC6792045; doi:10.1080/22221751.2019.1673135)

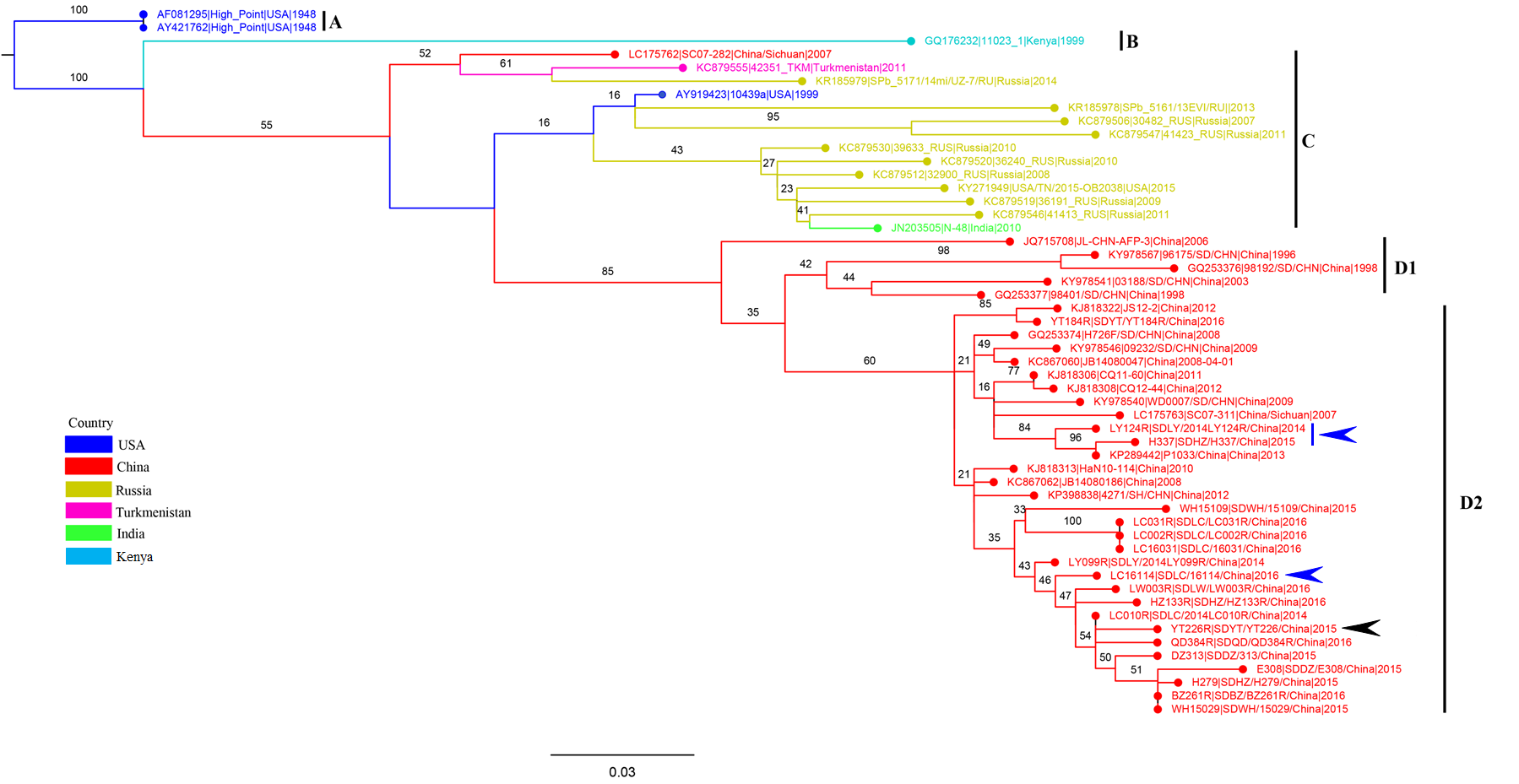

Supplement: Supplemental Material [file TEMI_A_1673135_SM2939.zip › Fig. S1.tif]

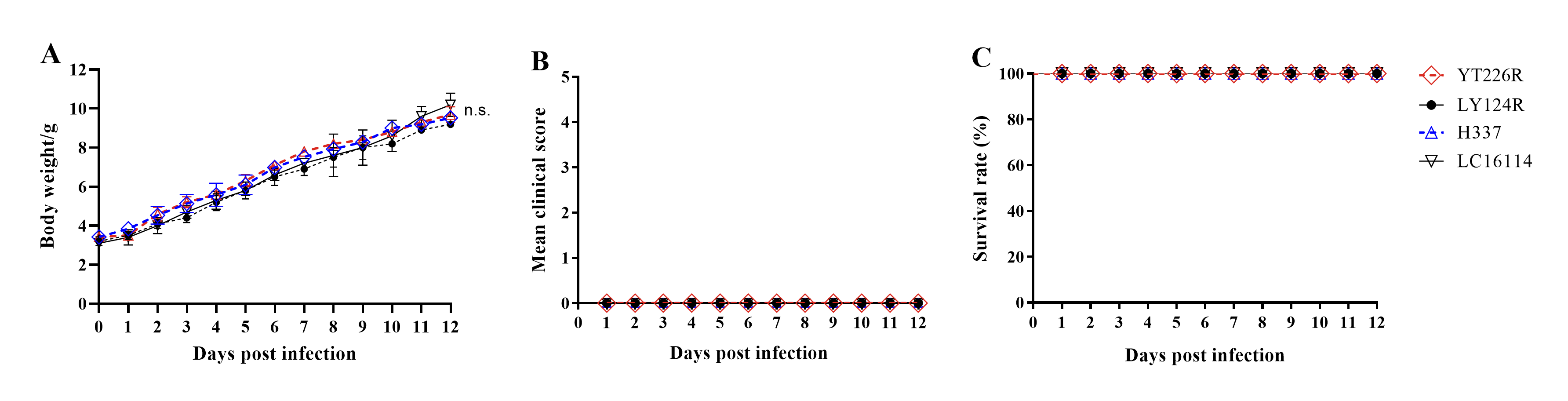

Supplement: Supplemental Material [file TEMI_A_1673135_SM2939.zip › Fig. S2.tif]
